# Evaluating the impact of a parent champion model on bronchiolitis hospitalisation rates: a difference in differences study

**DOI:** 10.1136/archdischild-2025-328671

**Published:** 2025-10-22

**Authors:** Alice R Lee, Pieta Georgina Schofield, Olufemi Olajide, Oyebisi Osuolale, Elizabeth Camacho, Debi McAndrew, Iain Buchan, Benjamin Barr, Daniel B Hawcutt, Ian P Sinha

**Affiliations:** 1 Innovation and Research, Alder Hey Children’s NHS Foundation Trust, Liverpool, UK; 2 Institute of Life Course and Medical Science, University of Liverpool, Liverpool, UK; 3 Department of Public Health Policy and Systems, Institute of Population Health, University of Liverpool, Liverpool, UK; 4 Innovation Hub, Alder Hey Children’s NHS Foundation Trust, Liverpool, UK; 5 Children’s services, Liverpool City Council, Liverpool, England, UK; 6 NIHR Alder Hey Clinical Research Facility, Alder Hey Children's Hospital, Liverpool, England, UK; 7 Department of Women’s and Children’s Health, University of Liverpool, Liverpool, UK; 8 Department of Respiratory Medicine, Alder Hey Children’s Hospital, Liverpool, England, UK

**Keywords:** Child Health Services, Healthcare Disparities

## Abstract

**Background:**

Bronchiolitis and its outcomes have strong socioeconomic determinants. In Liverpool, a city with high deprivation and above-average bronchiolitis hospitalisations, we implemented the Respiratory Parent Champions in the Community intervention. Parent Champions were provided with training from paediatricians and community organisations. They delivered peer support and education around bronchiolitis and modifiable risk factors in local children’s centres.

**Methods:**

Using anonymised linked electronic health records for children born in Cheshire and Merseyside 2016–2023, we used inverse probability of treatment weighted difference-in-differences methods to compare the change in monthly bronchiolitis admissions after the intervention was introduced in the intervention cohort to the change in admissions in children living in other wards in the region that had not received the intervention, adjusting for differences between populations.

**Findings:**

Eight Respiratory Parent Champions conducted 18 050 family contacts across 16 wards in Liverpool over a 24-month period. The intervention was associated with a reduction in emergency admissions of 128 per 100 000 children per month (95% CI 43 to 214, p<0.01), in comparison to the non-intervention cohort. This equates to a 23.8% reduction in admissions or a total of 107 (95% CI 36 to 179) admissions saved per year in the intervention cohort. The preliminary estimated cost of yearly admissions saved was £219 243, with a net saving of £101 283 per annum (−£44 196 to +£248 811) after accounting for the cost of employing Parent Champions.

**Interpretation:**

This study highlights the potential of a targeted community-led, co-produced intervention to alleviate healthcare burdens and address socioeconomic inequalities in infant respiratory illnesses.

**WHAT IS ALREADY KNOWN ON THIS TOPIC**

**WHAT THIS STUDY ADDS**

- This difference-in-differences study evaluated the impact of a Respiratory Parent Champions programme on monthly bronchiolitis hospitalisation rates for intervention wards, finding that it led to a 23.8% reduction in monthly bronchiolitis hospitalisation rates. This reduction occurred in socioeconomically deprived areas at a time when inequalities in health were widening across the UK due to the effects of the COVID-19 pandemic and the subsequent cost-of-living crisis. Our findings contribute to evidence that place-based and peer-led interventions can improve child health outcomes in deprived areas.

**HOW THIS STUDY MIGHT AFFECT RESEARCH, PRACTICE OR POLICY**

- This study demonstrates the impact of community-based, co-produced interventions such as Respiratory Parent Champions in the Community in reducing infant emergency respiratory admissions and health service pressures. This is, to our knowledge, the first evaluation of a community peer support intervention aimed at reducing bronchiolitis hospitalisations, using causal methods. Similar approaches should be considered in comparable populations, alongside research to explore long-term impacts and cost-effectiveness to inform broader policy implementation.

## Introduction

 Early childhood exposures and illness are increasingly recognised as important determinants of long-term respiratory health, particularly among those experiencing socioeconomic disadvantage.^1 2^ These early-life inequalities contribute to persistent health disparities and represent a significant burden for healthcare systems. In the UK, where child health outcomes are poorer than in many comparable countries,^3^ inequality in hospitalisation across the life course costs the National Health Service (NHS) approximately £4.8 billion each year.^4^

**Figure 1:**
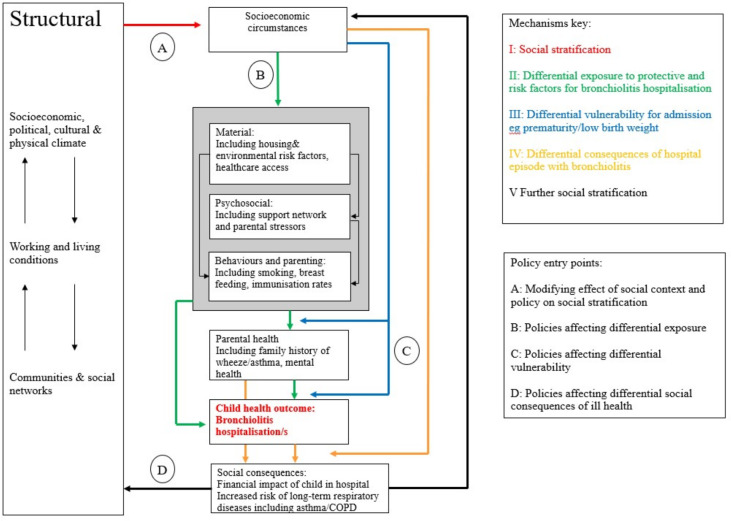
Adapted Diderichsen conceptual model demonstrating pathways to inequalities in bronchiolitis admission.^9^ COPD, chronic obstructive pulmonary disease.

One common and clinically significant illness in early childhood is bronchiolitis, a viral respiratory infection in infancy which is commonly caused by respiratory syncytial virus (RSV) and can lead to lasting damage to the developing lungs.^5^ In England, one in three infants will develop bronchiolitis,^6^ with well-described socioeconomic disparities in both outcomes and risk factors.^7 8^ Using an adapted Diderichsen model,^9^ these disparities can be understood as pathways linking broader social determinants, such as political and community structures and living conditions, to health outcomes. Risk factors for bronchiolitis hospitalisation, including premature birth, formula feeding and exposure to adverse environments,^58 10^,^12^ illustrate how these pathways contribute to inequalities (see figure 1).

Bronchiolitis hospitalisation rates had been rising steadily until the COVID-19 pandemic in 2020,^13^ when they sharply declined.^14^ Following the easing of pandemic restrictions in 2021–2022, many countries, including England, experienced a ‘viral surge’ marked by atypical seasonality and increased healthcare demand for bronchiolitis and related illnesses.^14^,^16^ This was expected to disproportionately affect children from the most socioeconomically deprived backgrounds, given the exacerbation of pre-existing social inequalities during the cost-of-living crisis that emerged in the later stages of the pandemic.

The adapted Diderichsen model in figure 1 can also be used to identify key points for intervention to mitigate the risk of widening inequalities in bronchiolitis outcomes. The model emphasises the importance of addressing broader structural drivers of social stratification, as well as differential exposures and vulnerabilities at individual and community levels.^9^ Historical place-based initiatives like Sure Start^17^ exemplify efforts to address these determinants. The programme successfully reduced inequalities in infant mortality and child development, although results for service utilisation and other health outcomes were mixed.^17^,^22^ Currently, local authority-funded children’s centres serve as community hubs offering free services to families with children under five. Targeted peer support has also been effective in addressing differential exposures, vulnerabilities and consequences for chronic conditions^23^,^25^ and behaviours such as smoking cessation^26^ and breastfeeding.^27^ However, to our knowledge, such strategies have not been evaluated for their potential to reduce inequalities in bronchiolitis hospitalisation.

## Objectives

Liverpool experiences high levels of deprivation^28^ and persistently higher than average rates of bronchiolitis hospitalisations and associated risk factors.^13^ In anticipation of a postpandemic viral surge, we implemented a parent-led intervention embedded within the existing children’s centre infrastructure. The Respiratory Parent Champions in the Community programme aimed to empower parents with knowledge about child respiratory health, rapidly and at scale, and was well received by parents and community stakeholders.^29^ This study evaluates whether the programme reduced emergency hospitalisation rates for bronchiolitis and related illnesses.

### Intervention

**Figure 2:**
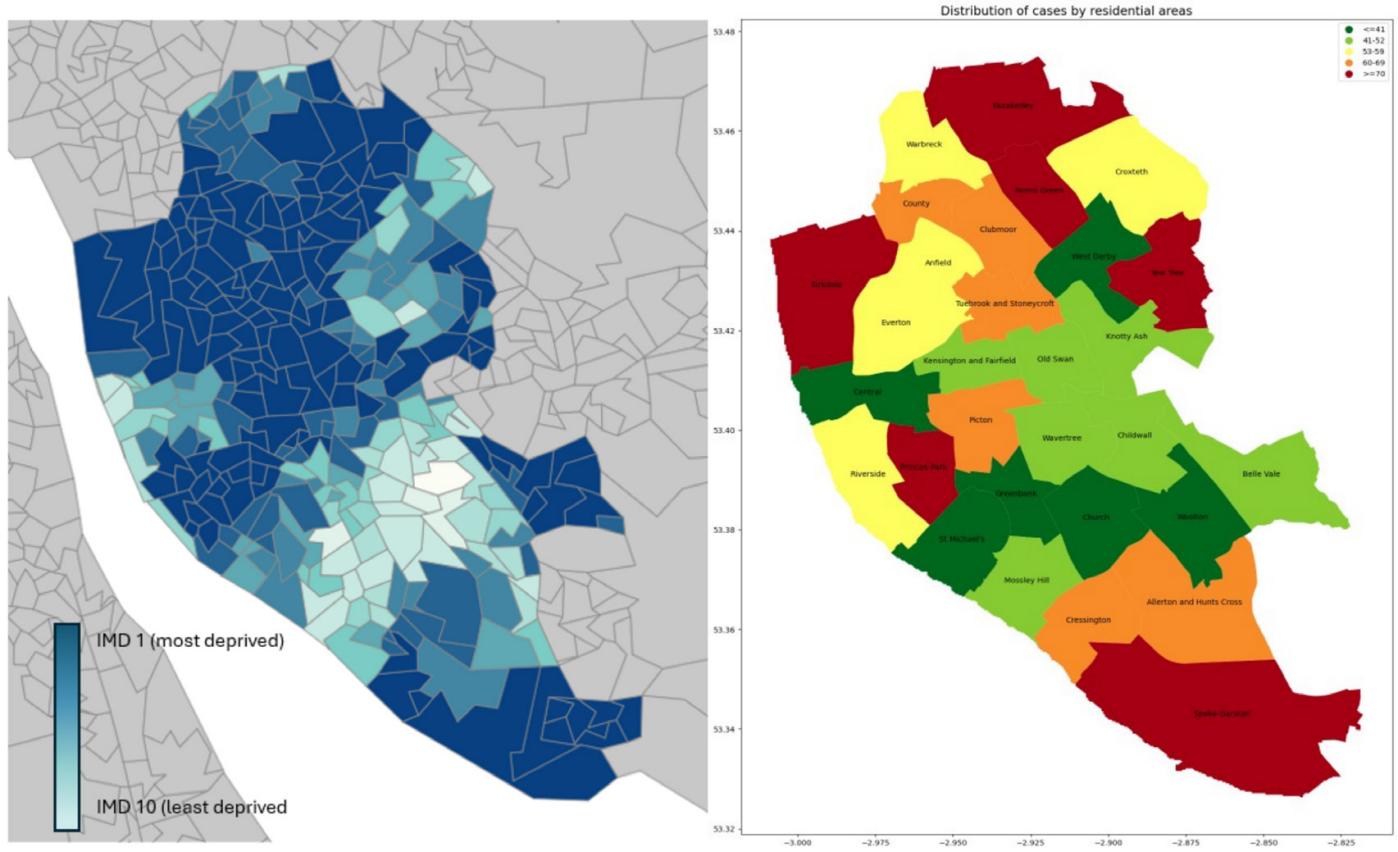
(A) Map of Index of Multiple Deprivation across Liverpool from the Ministry of Housing, Communities and Local Government and (B) Heat map for bronchiolitis hospitalisations 2017–2019 (taken preintervention and pre-COVID).

The Respiratory Parent Champions initiative employed local mothers to provide advice and peer support aimed at improving infant lung health.^29^ 16 electoral wards were selected as intervention areas based on hospital utilisation and socioeconomic geographic data (figure 2). Wards were also used as the unit of analysis, as smaller geographic areas could not be easily aligned with children’s centre catchment populations. To minimise problems of geographical boundary leakage, all data were analysed at the ward level.

The intervention aimed to address key pathways linking social determinants to bronchiolitis outcomes, as outlined in the adapted Diderichsen model. It targeted Pathway I by addressing social stratification, supporting families to access poverty reduction schemes such as the Citizen’s Advice on Prescription programme^30^ and providing monetary vouchers for essentials like fuel and food. It also addressed Pathway II, reducing differential exposure to risk through education, peer support and social prescribing. This included promoting breastfeeding, smoking cessation and immunisation uptake, offering psychosocial support, and signposting families to housing assistance and healthcare services. Parent Champions also worked to raise community awareness of bronchiolitis, providing education on prevention, self-management and navigating healthcare services (see the TIDieR tool in online supplemental file 2 for further details).

Eight Parent Champions—mothers reflective of the local community (50% non-white British; 50% multilingual)—were recruited from among recent children’s centre service users and employed by Alder Hey Children’s Hospital on NHS Band 3 contracts. The intervention launched in January 2022.

Parent Champions deliver the intervention through existing children’s centre infrastructure, offering individual and group support via home visits, children’s centre activity groups and clinics, and community outreach events. The programme and accompanying educational materials were co-produced by Parent Champions, clinicians and children’s centre staff. Parent Champions were trained, in response to their self-identified needs, by healthcare professionals and community organisations, including breastfeeding charities, advocacy and fire services.

### Design

The evaluation of the Respiratory Parent Champions programme was conducted as an opportunistic quasi-experimental study, using routinely collected data to generate timely insights.

#### Data sources

A population cohort for healthcare utilisation was built in the Combined Intelligence for Population Health Action (CIPHA) Secure Data Environment, now known as Data into Action (https://dataintoaction.cheshireandmerseyside.nhs.uk). CIPHA holds near real-time, pseudonymised general practitioner (GP), inpatient, outpatient, community and emergency care data for the Cheshire and Merseyside Integrated Care System (an NHS region comprising 2.6 million of the general population) from 2010 onwards for the purposes of care, planning and research.

Perinatal Hospital Episode Statistics (HES) data were used to identify children born between 2016 and 2023 in Cheshire and Merseyside. Records were linked with HES data for emergency hospitalisations (inpatient stays) between January 2018 and December 2023, as well as GP and community service datasets for relevant independent variables. As the cohort was defined through HES, children without a recorded perinatal hospital episode were excluded from the analysis.

#### Intervention cohort

The intervention cohort was defined as children aged 0–24 months living in the 16 intervention wards during the intervention period (January 2022–December 2023). The control cohort was defined as children aged 0–24 months living in the other 220 non-intervention wards across Cheshire and Merseyside.

#### Outcome measures

Our primary outcome was the monthly rate of emergency hospitalisation for bronchiolitis and bronchiolitis-type illnesses. Monthly data intervals were chosen to capture seasonal variation as well as improve statistical power for analysis. International Classification of Diseases 10th edition (ICD-10) codes for acute bronchiolitis, viral lower respiratory tract infection and viral wheeze were chosen for our health outcomes (online supplemental file 1). We included ICD-10 codes beyond ‘acute bronchiolitis’ to recognise common diagnostic uncertainties, particularly between bronchiolitis and viral wheeze for infants aged 1–2 years.

#### Statistical analysis

To assess the average treatment effect on the treated (ATT) for infants living in our intervention wards, we used a difference-in-differences model with inverse probability of treatment weighting.

### Difference-in-differences (DID)

The DID approach estimates the causal effect of an intervention by comparing the change in outcomes in the intervention cohort relative to the change in the control cohort. By using a comparison both within and between groups, DID isolates the intervention effect by accounting for both trends over time that affect both groups (eg, the COVID-19 pandemic) and time-invariant differences between groups that could otherwise confound findings.^31^

The parallel trends assumption (PTA), which is a fundamental assumption for DID analyses, was that the difference in rates of hospitalisation between our intervention and control cohorts would have remained constant without the intervention.

We used linear regression models within a DID framework to evaluate the intervention’s impact on monthly bronchiolitis admission rates. The regression model formula for our analysis was as follows:

Y_it_=β0+ β1*time_period_t_+β2*intervention_status_i_+β3(time_period_t_*intervention_status_i_)+ϵ_it_

where Y_it_ is the observed value (admission rate) for unit i at time t, β3 captures the ATT, and ϵ_it_ represents unexplained variability. Clustered SEs accounted for within-group/individual and time-related correlations.

### Inverse probability of treatment weighting (IPTW)

IPTW was used to adjust for potential confounding by balancing observed differences between the two groups that could have influenced admission trends in the absence of the intervention. As intervention exposure was assigned by electoral ward, IPTWs were also calculated at the ward level. Propensity scores were derived from a logistic regression predicting the probability of being in an intervention ward, using predictors of preintervention hospitalisation trends, percentage of white British ethnicity and population characteristics associated with bronchiolitis admissions (Index of Multiple Deprivation score, percentage of premature births at<37 weeks gestation; online supplemental file 1). These scores were used to calculate IPTWs for the control cohort, which were incorporated into the DID model.^32^ By balancing these covariates between intervention and control groups, this approach reduces bias from differential pre-intervention admission trends related to these factors.

We conducted sensitivity analyses to evaluate the robustness of our model choice and data input selection (online supplemental file 1). For model choice, we ran two further DID models: one without IPTW and another using a Poisson regression, comparing the marginal effects to those of the linear regression outputs. In our main analysis, we used patient-level data to capture variability and heterogeneity across individuals within cohorts and used the full dataset to maximise statistical power and precision. To assess sensitivity to data granularity, we reran the model using aggregated data. Robustness checks included fidelity to the PTA by (1) visual inspection and (2) statistical analysis for trends in differences of hospitalisation rates between the two groups in the preintervention period (online supplemental file 1). Visual trends during the COVID-19 pandemic confirmed that our data do not violate the common shocks assumption. Results are presented as main effects with 95% CI unless otherwise stated. All statistical analyses were computed using R V.4.2.1.

#### Preliminary cost analysis

As our admission data were not disaggregated by severity, we calculated a weighted mean unit cost using national data that capture the distribution of admission severity for each ICD-10 diagnostic code. This method ensures that cost estimates reflect national variation in case complexity and length of stay, providing a representative estimate of unit costs across the country.

Each ICD-10 code corresponds to multiple currency codes within the NHS National Schedule of Reference Costs, accounting for differences in complexity and duration of hospital stays for the same diagnosis.^33^ We calculated the weighted mean unit cost by aggregating costs across all relevant currency codes. For each currency code, we recorded the number of finished consultant episodes (FCEs) and the national average unit cost. The total cost per code was obtained by multiplying the number of FCEs by the unit cost. Summing these total costs across all codes and dividing by the total number of FCEs yielded a weighted mean unit cost.

This analysis is intended as a pragmatic, indicative assessment of potential cost implications for a hospital employing the Parent Champions, rather than a complete health economic evaluation. Our aim was to provide context on the possible financial impact from the provider’s perspective, based on estimated reductions in admissions. Net annual savings were calculated as:


$$\begin{array}{l} Net\;annual\;savings=\;(weighted\;mean\;cost\;per\;admisssion\times \;estimated\;reduction\;in\;admissions)\\ \;-yearly\;cost\;of\;employing\;Parent\;Champions \end{array}$$

## Results

Our intervention cohort was significantly more socioeconomically deprived and ethnically diverse than the Cheshire and Merseyside average, with a higher rate of prematurity. However, when weighting for IPT, these differences were accounted for (table 1 and online supplemental file 1). Between January 2022 and the end of December 2023, the Parent Champions had 18 050 contacts with families in these wards through direct outreach or Children’s Centre sessions.

**Table 1:** Population demographics for Cheshire and Merseyside infants

	Intervention areas	Non-intervention areas	
Group	n=25 565	n=179 413	P value^*^
Sex			
Male	51%	51%	0.2
Female	49%	49%	
Deprivation			
Index of Multiple Deprivation Deciles^†^	1 (1, 1)	4 (2, 8)	<0.001
Ethnicity			
White British	52%	69%	<0.001
Prematurity			
Premature (<37 weeks)	8.40%	7.60%	<0.001

* χ2 test; Mann-Whitney test

† %; median (quartiles 1 and 3).

### Comparison of bronchiolitis hospitalisation rates

**Figure 3:**
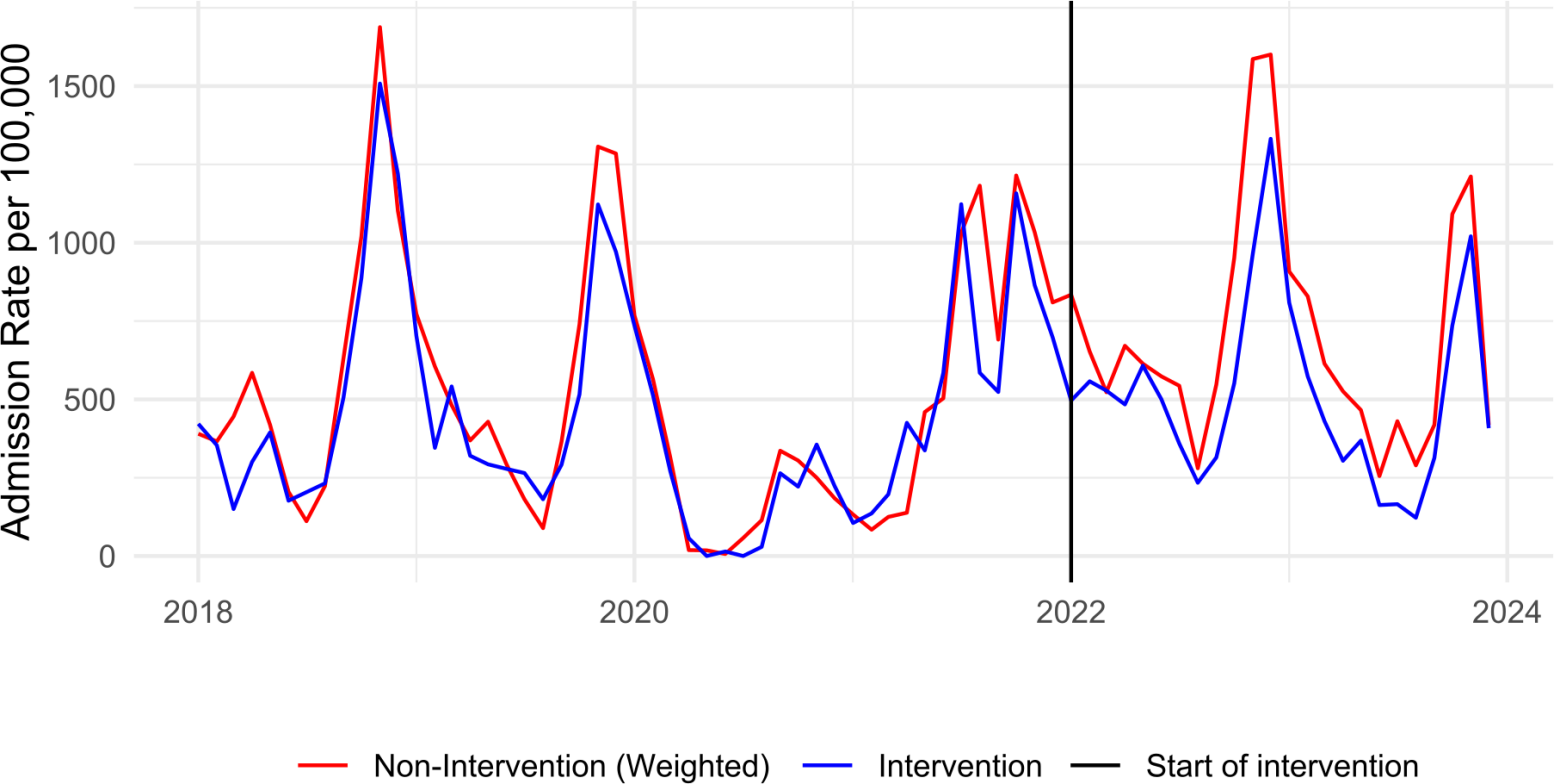
Monthly hospitalisation rates for bronchiolitis and bronchiolitis-type illnesses in toddlers aged 0–2 years in intervention and inverse probability weighting-adjusted non-intervention cohorts in Cheshire and Merseyside, January 2018–December 2023

Monthly hospitalisation rates for our intervention cohort and IPTW-adjusted rates for the non-intervention cohort between January 2018 and December 2023 are shown in figure 3. There were a total of 21 903 hospitalisations for bronchiolitis and related illnesses across Cheshire and Merseyside, with 7.5% of children having ≥1 hospitalisation in the first 2 years of life. The mean age at hospitalisation was 41 weeks, and the most common age at hospitalisation was 8 weeks. The average monthly rate of hospitalisation for both groups was 579 per 100 000, with expected seasonal variation. Hospitalisation rates dropped to 11 per 100 000 in June 2020 after the introduction of pandemic restrictions on mixing, with a summer and winter peak in 2021 before returning to prepandemic patterns in 2022. DID analysis for hospitalisation rates in the preintervention period showed no significant difference in trend between our intervention and non-intervention cohorts (online supplemental file 1). From the introduction of Parent Champions in January 2022, the intervention cohort had persistently lower monthly hospitalisation rates, with an average monthly hospitalisation of 524 per 100 000 (95% CI 473 to 574) in comparison to an average of 691 per 100 000 (95% CI 668 to 714) in the non-intervention wards.

Our IPT-weighted DID model demonstrated a reduction in bronchiolitis hospitalisations following the introduction of the intervention, relative to the change in the non-intervention cohort. The interaction coefficient for intervention×time, representing the ATT, showed a reduction in hospitalisation rates of 128 per 100 000 (95% CI −43 to −214, p<0.005). SEs were clustered by individuals and date.

To aid interpretability, this result can be presented as a 23.8% (95% CI 8% to 39.9%) change relative to the average preintervention monthly rates in the treatment group (537 per 100 000). The baseline rate excludes the COVID-19 period (from February 2020 onwards), as including this period would artificially lower the mean and inflate the estimated per cent reduction. This is equivalent to a total of 107 (95% CI 36 to 179) fewer admissions per year than would have otherwise been the case in the absence of the intervention. Additional analyses using aggregated data, unweighted data and sensitivity tests employing Poisson regression yielded similar results (online supplemental file 1).

### Cost analysis

The weighted mean unit cost as per our ICD-10 codes was £2049. Estimated net annual savings were calculated by multiplying the DID-derived estimate of admissions avoided by the weighted mean unit cost and subtracting the annual cost of employing the parent champions.

Based on an estimated 107 (95% CI 36 to 179) admissions prevented per year, the gross savings total £219 243 (£73 764–£366 771). The cost of running the model was £117 960 per year. After accounting for this, the average estimated net yearly savings to the NHS amounts to £101 283 (−£44 196 to +£248 811).

## Discussion

Our study shows that the Respiratory Parent Champions in the Community programme was associated with a 23.8% reduction in monthly bronchiolitis hospitalisation rates in the target population. This reduction occurred in socioeconomically deprived areas of the region at a time when (1) health inequalities were widening across the UK following the COVID-19 pandemic and cost-of-living crisis and (2) national rates of bronchiolitis admissions were elevated compared with prepandemic levels.^34^

Our study demonstrates the impact of a place-based, co-produced preventive intervention—targeting the pathways to early childhood respiratory health inequalities identified in the adapted Diderichsen model in figure 1—in reducing hospitalisation rates for bronchiolitis. This is, to our knowledge, the first study showing a multifaceted (and multiply targeted) peer support correlating to reduced hospitalisation rates in an infant population. It is, however, in keeping with previous work demonstrating the effectiveness of peer support for chronic conditions.^18 19^ Our findings also demonstrate a rapid impact on healthcare utilisation when deploying a community-based initiative, in comparison to previous evidence which has yielded mixed results for the impact of such interventions in high-income countries.^24 26^ Similar programmes could be adapted and implemented in other settings by leveraging existing community services that influence the social determinants of health and through meaningful co-production with local communities.

Given the quasi-experimental design of this study, there are inherent limitations to causal inference. However, while the possibility of unmeasured individual-level confounding cannot be fully excluded, we employed IPTW to adjust for population-level differences, particularly in socioeconomic deprivation and preintervention hospitalisation trends. Additionally, the DID approach mitigates bias from time-invariant confounders and from external shocks or trends that would have similarly affected both groups. At the small-area (electoral ward) level, there were no concurrent interventions or events that could explain the observed reductions in admission rates, and sensitivity analyses yielded consistent results.

Considering financial constraints in the NHS, a complete health economics analysis is important for an accurate assessment of cost-effectiveness. We adopted a pragmatic approach to provide a preliminary cost analysis, guided by available data, rather than conducting a full economic evaluation. Our analysis provides indicative insight but excludes potential benefits such as reductions in other healthcare utilisation (eg, emergency department attendances), parental work absences, long-term health outcomes and social return on investment. Longitudinal data would enable evaluation of the intervention’s impact on long-term respiratory health outcomes, particularly in the context of the recent introduction of the maternal RSV vaccine. Likewise, we did not account for all costs associated with the programme, such as increased costs to community services or initial materials. No additional training costs were included; Parent Champions’ time was accounted for as an ongoing employment cost, reflecting ‘on the job’ training, while training provided by healthcare professionals and community organisations occurred within existing structures without charge. We acknowledge that these assumptions may not hold in other regions or settings.

## Conclusion

In a context where health inequalities and system pressures from acute respiratory illnesses represent major challenges for the NHS, our findings suggest that community-based interventions co-produced between the NHS and local community structures can reduce paediatric healthcare utilisation and alleviate service pressures among targeted populations. This approach warrants consideration in policy strategies addressing winter pressures and health inequalities. While the intervention was delivered through existing children’s centre infrastructure, highlighting the importance of such place-based assets, its core components could be adapted for scale in areas with equivalent community platforms. Further research is needed to assess long-term impacts on respiratory health, and data-driven implementation can support evaluations of cost-effectiveness and guide resource allocation. If replicated elsewhere, maintaining co-production with communities will be essential to ensure contextual relevance and maximise effectiveness.

### Role of the funding sources

Initial funding for the intervention was awarded through the NHS England Voluntary, Community and Social Enterprise (VCSE) health and well-being fund, with further support obtained through Liverpool City Council and Alder Hey Children’s Charity. Funding for a clinical fellow was secured through the VCSE award and through Health Data Research UK (HDRUK).

Quantitative evaluation was undertaken by the team with support from the University of Liverpool and Liverpool City Region Civic Data Cooperative (CDC). ARL is supported through the HDRUK rapid grant for data science to inform the NHS compound winter pressure policy response, and ARL and OO are both supported by the HDRUK Research Driver Programme: Inflammation and Immunity. PS, BB and IB are supported by the National Institute for Health Research Applied Research Collaboration North-West Coast. IB is supported by the NIHR as a senior investigator. The views expressed in this publication are those of the author(s) and not necessarily those of the National Institute for Health Research or the Department of Health and Social Care.

Neither the funders of the study nor the intervention had any role in study design, data collection, data analysis, data interpretation or writing of the report.

## Supplementary material

10.1136/archdischild-2025-328671 online supplemental file 1

10.1136/archdischild-2025-328671 online supplemental file 2

## Data Availability

Data may be obtained from a third party and are not publicly available. No data are available.
