# Supplementary material for: Evaluating the impact of a parent champion model on bronchiolitis hospitalisation rates: a difference in differences study
Source: Arch Dis Child. 2025 Oct 22;111(2):e328671. doi: 10.1136/archdischild-2025-328671 (PMC12911602; doi:10.1136/archdischild-2025-328671)
Supplement: online supplemental file 1 [file archdischild-111-2-s001.doc]

**Supplementary Material: Evaluating the impact of a Respiratory Parent Champion model on bronchiolitis hospitalisation rates: a difference in differences study**

**Appendix 1: ICD 10 codes**

Table 1: ICD 10 codes used to identify bronchiolitis or bronchiolitis type illnesses 0-2 years age

| CODE | DESCRIPTION |
| --- | --- |
| **J21** | **Acute bronchiolitis** |
| J21.0 | Acute bronchiolitis due to respiratory syncytial virus |
| J21.1 | Acute bronchiolitis due to human metapneumovirus |
| J21.8 | Acute bronchiolitis due to other specified organisms |
| J21.9 | Acute bronchiolitis, unspecified |
| **J12** | **Viral pneumonia, not elsewhere classified** |
| J12.0 | Adenoviral pneumonia |
| J12.1 | Respiratory syncytial virus pneumonia |
| J12.2 | Parainfluenza virus pneumonia |
| J12.3 | Human metapneumovirus pneumonia |
| J12.8 | Other viral pneumonia |
| J12.9 | Viral pneumonia, unspecified |
| **J22** | **Unspecified acute lower respiratory infection** |
| **R06.2** | **Wheezing** |

**Appendix 2: Inverse Probability Weighting**

The target areas receiving the Respiratory Parent Champion intervention were selected based on baseline demographic differences compared to non-intervention areas across Cheshire & Merseyside. Propensity scores, representing the probability that a unit would be assigned to the treatment group based on baseline characteristics, were therefore calculated for non-intervention wards to account for these differences (1).

Baseline observations were chosen due to increased risk of hospitalisation, and to recognise key differences in our intervention wards: median IMD score, percent of premature births (<37 weeks gestation) and percent of population with non-White British ethnicity. Our intervention areas have significantly higher levels of ethnic diversity compared to the Cheshire and Merseyside average, where 90% of the population identified as White British according to the 2021 Census (2). This demographic distribution posed challenges for analysing data for individual ethnic minority groups due to the relatively small numbers within each group. To maintain statistical power and ensure robust analysis, all non-White British ethnicities were therefore grouped into a single "Non-White British" category.

These propensity scores were then used to create a comparable control group through the application of inverse probability of treatment weighting (IPTW). This approach generates a pseudo-population from the non-intervention wards in Cheshire & Merseyside, ensuring that the baseline characteristics of the control group closely mirrored those of the intervention group for use in the analysis (3).

TABLE 2: Balance for intervention and non-intervention cohorts after Inverse Probability Weighting

|  | Non-Intervention | Intervention | p Value |
| --- | --- | --- | --- |
| Percent premature (mean (SD)) | 8.46 (1.44) | 8.34 (0.98) | 0.739 |
| Median IMD (mean (SD)) | 1,973.45 (2,200.40) | 1,585.25 (1,767.97) | 0.481 |
| Percent White British (mean (SD)) | 62.18 (13.96) | 54.56 (19.24) | 0.156 |
| Admission rate 2018 (mean (SD)) | 584.87 (198.69) | 547.36 (143.39) | 0.424 |
| Admission rate 2019 (mean (SD)) | 564.66 (169.26) | 505.58 (203.36) | 0.282 |
| Admission rate 2020 (mean (SD)) | 255.25 (119.38) | 237.17 (119.92) | 0.634 |
| Admission rate 2021 (mean (SD)) | 620.65 (195.13) | 579.17 (176.56) | 0.443 |

**Appendix 3: Sensitivity Analyses**

To test sensitivity to our model choice, we ran the linear OLS difference-in-differences model using non-IPTW weighted data and ran a Poisson regression difference in differences model. To test sensitivity to data granularity, we ran the difference-in-differences model at different levels of data aggregation.

*Difference-in-differences model using non-IPTW non-intervention cohort*

As discussed in Appendix 2, inverse probability of treatment weighting (IPTW) was implemented in our analysis to adjust for baseline differences between intervention and control groups. However, IPTW can increase the risk of type I errors, potentially leading to the incorrect rejection of the null hypothesis—that the intervention does not impact hospitalisation rates compared to non-intervention cohort (4). To address this concern, we conducted a sensitivity analysis by running the difference-in-differences model without IPTW for the control cohort.

TABLE 3: Difference-in-differences output using non – inverse probability weighted control cohort with standard error clustering for individuals and time

| Dependent variable | Effect on hospitalisation rate per 100,000 (95% CI) | P value |
| --- | --- | --- |
| Intervention x time | - 86.4 (25.8 ,147) | 0.005 |

The results of the non-IPTW weighted difference-in-differences model also indicate a statistically significant change in differences following the intervention.

Figure 1: Monthly hospitalisation rates for bronchiolitis and bronchiolitis-type illnesses in 0–2-year-olds in intervention and non-weighted non-intervention cohorts in Cheshire and Merseyside


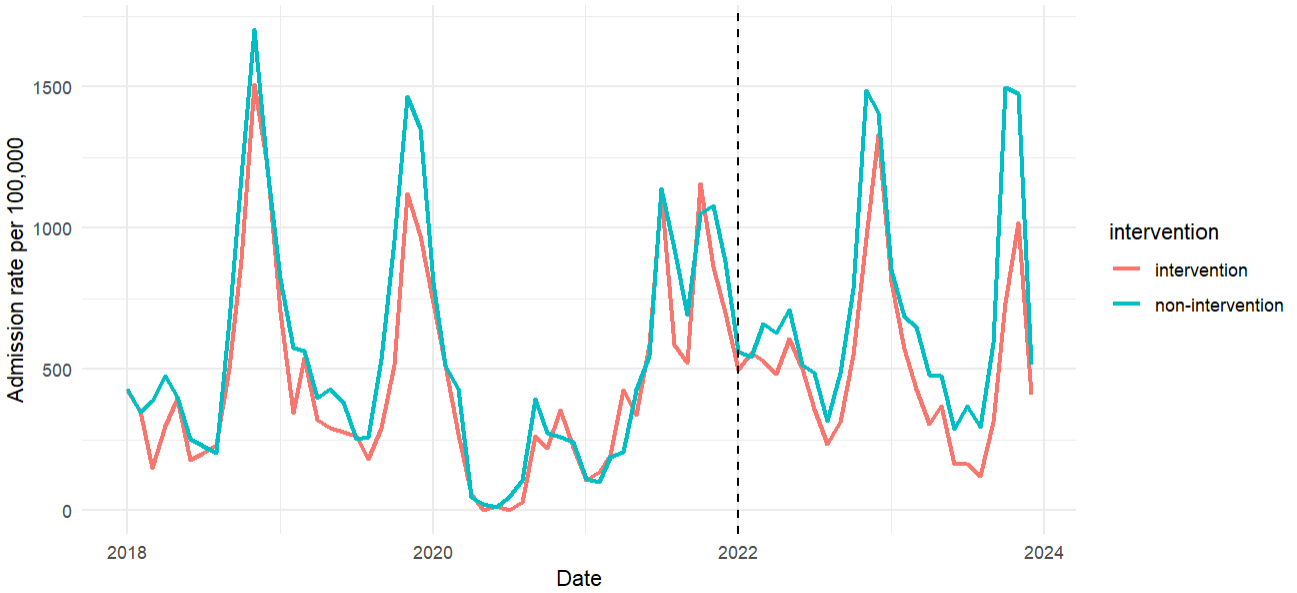


Figure 2: Annual hospitalisation rates for bronchiolitis and bronchiolitis-type illnesses in 0–2-year-olds in intervention and non-weighted non-intervention cohorts in Cheshire and Merseyside


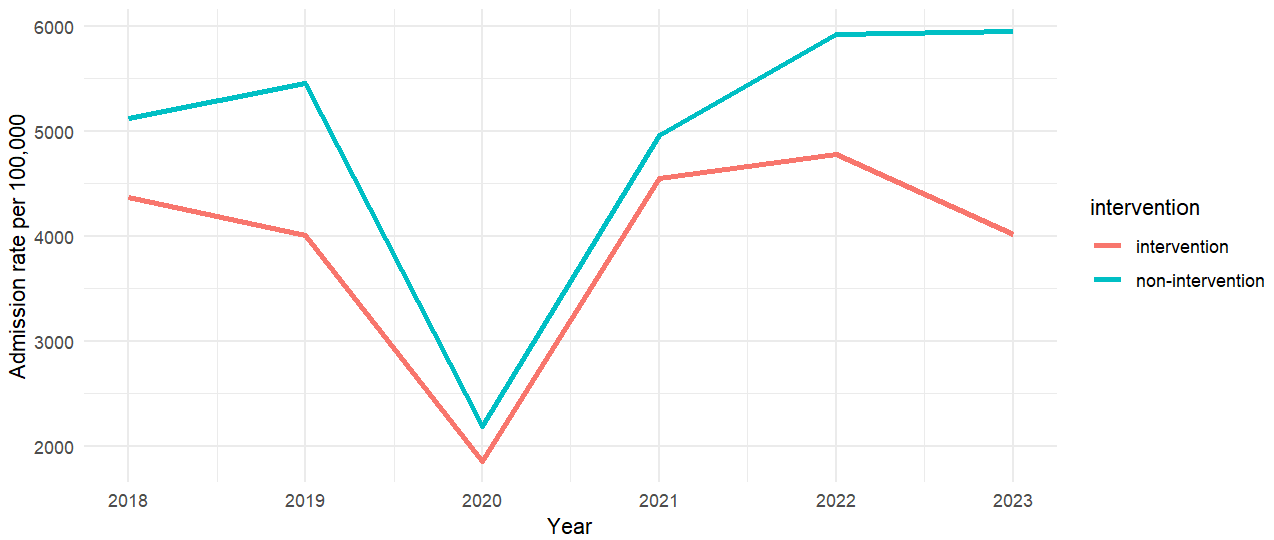


*Difference-in-differences model using a Poisson regression*

Conventional difference-in-differences analyses such as our model employs linear regression to estimate the average treatment effect on the treated (ATET) (5). While logistic regression is more commonly used for count or rate data, our outcome variable is the change in rate—a continuous measure—which makes linear regression a more suitable model for this analysis (6).

To ensure our findings were not influenced by the choice of a linear regression model, we performed a sensitivity analysis using a Poisson regression model for the difference-in-differences framework. In this analysis, Poisson regression was used to estimate the coefficient for the Intervention*Time interaction term, which we then used to calculate the marginal effect on hospitalisation rates.

Table 4: IPTW-weighted difference-in-differences model using Poisson regression

| Dependent variable | Estimated coefficient | P value |
| --- | --- | --- |
| Intervention x time | -0.187822 | 0.0097 |

Table 5: Predicted marginal effect model using the weighted difference-in-differences using Poisson regression

| Time | Intervention status | Predicted rate per 100,000 | 95% CI |
| --- | --- | --- | --- |
| 0 | Non-intervention | 509.19 | 474.96, 545.90 |
| 0 | Intervention | 449.44 | 396.06, 510.01 |
| 1 | Non-intervention | 700.63 | 639.75, 767.30 |
| 1 | Intervention | 512.52 | 454.99, 577.32 |

The estimated rate change difference using the predicted marginal effects from the Poisson regression model in Table 5 is 128 per 100,000. This result provides confidence that the findings from our linear regression difference-in-differences analysis are robust to our choice of model.

*Difference-in-differences models using aggregated data*

In our main analysis, we used individual child-level data. This level of granularity allows us to capture variability and heterogeneity across individuals within wards and use the full birth dataset to maximise statistical power and precision. However, because the intervention was implemented at the ward level, and the inverse probability of treatment weights (IPTW) were also calculated at the ward level, it is important to assess whether aggregating data to ward level affects the estimated impact of our intervention.

To test sensitivity to data granularity, we therefore re-ran the difference-in-differences model using ward-level aggregated data. By comparing the individual-level and ward-level analyses, we confirm that the observed treatment effects are robust to the level of data granularity:

Table 6: Difference-in-differences model outcomes using aggregated data to ward level

| Dependent variable | Effect on hospitalisation rate per 100,000 (95% CI) | P value |
| --- | --- | --- |
| Intervention x time | -150 (-238, -62.0) | 0.000914 |

*Indicates the change in hospitalisation rates at ward aggregated level in the intervention wards before and after the start of intervention compared to the change in hospitalisation rates in non-intervention wards, with standard error clustering for wards and date.*

**Appendix 4: Robustness Checks**

*Clustered standard errors*

In the main difference-in-differences formula, ϵ_it denotes the error term for unexplained variability. However, this relies on errors being independently distributed across observations (7).

In our data, error terms are unlikely to be independently distributed. This is due to the presence of multiple observations for the same individuals or groups (e.g., individuals at higher risk of admission) and temporal factors, including seasonality and the COVID-19 pandemic, affecting hospitalisation rates. To address this, we applied cluster-robust standard errors to account for within-group correlation at the individual level (ward level for our aggregated sensitivity analysis) and by time to account for temporal patterns and common shocks.

*Assessing the parallel trends assumption:*

To test the parallel trends assumption, we ran a difference-in-differences regression for the pre-intervention time period.

Table 7: IPTW weighted difference-in-differences outcome for hospitalisation rates in intervention and non-intervention areas for pre-intervention time period, with standard error clustering for individuals and time

| Dependent variable | Effect on hospitalisation rate (95% CI) | P value |
| --- | --- | --- |
| Intervention x time | 1.19e-7 (-1.44e-6, 1.20e-6) | 0.857 |

As the Intervention*Time interaction coefficient is not significant, this indicates that differences in bronchiolitis hospitalisations between our groups were stable in the pre-intervention time period, and therefore we can accept the parallel trend assumption.

References

1. Stuart, E. A., Huskamp, H. A., Duckworth, K., Simmons, J., Song, Z., Chernew, M. E., & Barry, C. L. (2014). Using propensity scores in difference-in-differences models to estimate the effects of a policy change. *Health Services and Outcomes Research Methodology*, *14*(4), 166–182.
2. Office for National Statistics (ONS), released 29 November 2022, ONS website, statistical bulletin, [Ethnic group, England and Wales: Census 2021](https://www.ons.gov.uk/peoplepopulationandcommunity/culturalidentity/ethnicity/bulletins/ethnicgroupenglandandwales/census2021)
3. Chesnaye, N. C., Stel, V. S., Tripepi, G., Dekker, F. W., Fu, E. L., Zoccali, C., & Jager, K. J. (2022). An introduction to inverse probability of treatment weighting in observational research. *Clinical Kidney Journal*, *15*(1), 14–20.
4. Xu, S., Ross, C., Raebel, M. A., Shetterly, S., Blanchette, C., & Smith, D. (2010). Use of Stabilized Inverse Propensity Scores as Weights to Directly Estimate Relative Risk and Its Confidence Intervals. *Value in Health*, *13*(2), 273–277.
5. Wing, C., Simon, K., & Bello-Gomez, R. A. (2018). Designing Difference in Difference Studies: Best Practices for Public Health Policy Research Keywords. *Annu. Rev. Public Health*, *39*, 453–469.
6. Rothbard, S., Etheridge, J. C., & Murray, E. J. (2023). A Tutorial on Applying the Difference-in-Differences Method to Health Data. *Current Epidemiology Reports*, *11*(2), 85–95.
7. Lee, Y. R., & Pustejovsky, J. E. (2023). Comparing Random Effects Models, Ordinary Least Squares, or Fixed Effects With Cluster Robust Standard Errors for Cross-Classified Data. *Psychological Methods*.
