# Supplementary material for: Evaluating the impact of a parent champion model on bronchiolitis hospitalisation rates: a difference in differences study
Source: Arch Dis Child. 2025 Oct 22;111(2):e328671. doi: 10.1136/archdischild-2025-328671 (PMC12911602; doi:10.1136/archdischild-2025-328671)
Supplement: online supplemental file 2 [file archdischild-111-2-s002.pdf]

TIDieR

Respiratory Parent Champions in the Community

Why:

The intervention was designed to address the socioeconomic determinants of bronchiolitis outcomes in Liverpool, where hospitalisation rates for bronchiolitis are higher than the national average. These elevated rates correlate with high levels of socioeconomic deprivation in the region.

By employing eight Respiratory Parent Champion model, Alder Hey Children's Hospital aimed to empower parents through:
1. **Raising Awareness of Bronchiolitis:** Educating families about the condition, its symptoms, and risk factors to improve early recognition and management at home.
2. **Improved Healthcare Access:** Guiding parents on when and how to seek appropriate healthcare, thereby reducing unnecessary hospital visits and admissions.
3. **Addressing Risk Factors:** Empowering families to address risk factors such as poor housing conditions, exposure to second-hand smoke, and hygiene practices that contribute to viral transmission.
4. **Promoting Protective Factors:** Supporting breastfeeding, nutrition, and immunisations, which play a protective role in respiratory health.

The intervention combines education, peer support, and social prescribing, creating a multifaceted approach that integrates with existing Children's Centre infrastructure. The ultimate goal is to reduce bronchiolitis-related hospitalisations by improving health literacy, addressing socioeconomic barriers, and fostering community resilience (see Direct Acyclic Graph).

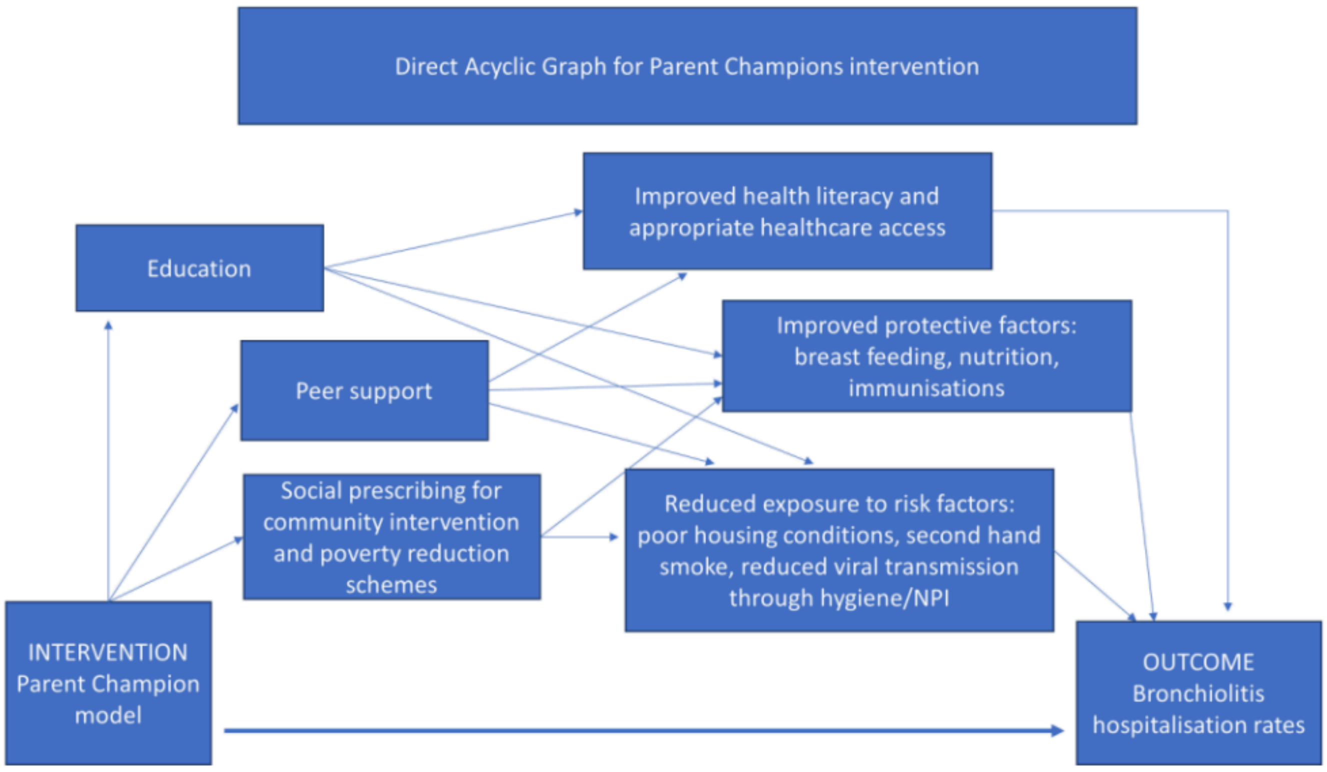

What (material):

The intervention utilised a variety of physical and informational materials to support both the training of respiratory Parent Champions and their delivery of interventions to families.

Training Materials:
Training was delivered by a range of health and social organisations across Liverpool, including Alder Hey Children's Hospital, Liverpool Women's Hospital, Shelter, Merseyside Fire & Rescue Services, Liverpool Bambis, Citizen's Advice, and local Children's Centres. Each organisation provided its own training resources and materials, ensuring a comprehensive knowledge base for Parent Champions.

Intervention Materials for Families:
Respiratory Parent Champions in the Community Two co-designed leaflets were developed in collaboration with Parent Champions: one explaining bronchiolitis and another outlining its key risk factors. Videos created by Parent Champions demonstrated respiratory symptoms and signs, utilising materials from Ask Sniff and the Healthier Together initiative. Multilingual videos provided by Bliss ensured accessibility for families from diverse linguistic backgrounds The Alder Hey symptom checker was promoted as an accessible, user-friendly tool for families to assess symptoms and seek guidance.

What (procedures):

The intervention is embedded within local children's centre infrastructure to deliver peer support, education, and social prescribing related to bronchiolitis and respiratory health. The procedures and activities undertaken by the respiratory Parent Champions are as follows:

Work Allocation: Parent Champions typically work 0.5 full-time equivalent (FTE) hours, except for one who works full time.

Outreach Activities: Parent Champions conduct on average 3 outreach sessions per week, which include home visits and telephone calls based on antenatal lists provided by the local authority (LA). These sessions are designed to engage families with new babies in their homes, offering tailored support and signposting to Children's Centres and other local services.

Group Support: Parent Champions actively support on average 2-3 parent-and-baby groups at Children's Centres per week, providing informal education and support around bronchiolitis and wider determinants of respiratory health to Children's Centre users.

Presence at Community Hubs: Parent Champions staff and support desks and waiting rooms for Children's Centre services including health visitor clinics, neonatal audiology, maintaining a poster board and being available to offer information and assistance to families. Each champion supports 0-2 clinics per week.

Community Education: Larger-scale education efforts are carried out by distributing leaflets and informational materials to local nurseries, pharmacies, GP surgeries and supermarkets. Parent Champions also engage with the broader community through hosting stands at events such as Breastfeeding Week, Baby Week, and Family Hub openings.

Signposting and Referrals: Parent Champions connect families to relevant local services and resources. They provide tailored signposting and referrals to the organisations involved in their training including the Liverpool Citizens Advice on Prescription scheme. Additionally, they refer families to poverty reduction schemes, including access to groceries and fuel vouchers, food pantries, and baby pantries.

These procedures are designed to enable a comprehensive, community-centered approach to providing respiratory health education and support for families.

Who provided:

The Parent Champions were recruited from local communities through their respective Children's Centres. They were all mothers who had previously used the services of the centres. No formal qualifications were required; instead, recruitment criteria focused on lived experience. The job was advertised for parents rather than mothers, but only mothers applied. Parent Champions were all hired on Band 3 contracts with Alder Hey Children's Hospital.

The training provided to Parent Champions was tailored to address known risk factors for bronchiolitis and respiratory health inequalities in early childhood. Training content was informed by the needs identified by the Parent Champions themselves and was co-designed in collaboration with local organisations and service providers:

Bronchiolitis teaching sessions were conducted by clinicians from Alder Hey Children's Hospital, supplemented with educational resources from the Healthier Together initiative.
Housing and Safety training was provided in partnership with Liverpool Shelter and Merseyside Fire & Rescue Services, focusing on housing-related health risks and tenancy rights.
Breastfeeding support training was conducted by Liverpool Bambis breastfeeding charity, equipping PCs to offer guidance on breastfeeding.
Smoking cessation guidance training was provided by both clinicians from Alder Hey Children's Hospital and Liverpool Women's Hospital midwifery service.
The Liverpool Perinatal Scheme Leader from Citizen's Advice provided education around their services and referral pathways for poverty reduction schemes.
Online e-learning training completed as mandated for Alder Hey staff
Additional training on schemes aimed at poverty alleviation, including voucher support, was coordinated through Children's Centres.

How (mode of delivery; individual or group):

The Parent Champions delivered the intervention through a combination of individual interactions, small group sessions, and wider community outreach. Each mode of delivery was tailored to the setting and audience.

Individual Family Interaction:
Telephone calls and home visits, guided by antenatal lists provided to Children's Centres from the Local Authority, conducted by Parent Champions (each Parent Champion would typically run home visit lists 2–4 times per week). Informal meetings occurred during waiting room and desk support sessions at Children's Centres, where Parent Champions engaged with families attending baby clinics or sessions.
Small Group Sessions:
Parent Champions facilitated on average 3 small group sessions at Children's Centres, including activities like stay-and-play, baby massage, and other parent-and-baby groups. They delivered educational content to the group while also engaging in individual conversations to address specific concerns.
Wider Community Outreach:
Parent Champions participated in larger community events, setting up stalls and stands to engage with families. These events took place at key community hubs, including libraries, GP surgeries, nurseries, and pharmacies. They distributed informational materials, answered questions, and provided peer support during these outreach activities.
Delivery Features:
Across all modes of delivery, Parent Champions shared physical materials such as leaflets and videos, provided education, and offered peer support. The sessions were interactive and tailored to the families' needs, ensuring accessible, face-to-face engagement in most instances.

Where:

The 16 Children's Centre locations were chosen based on Business Intelligence data on bronchiolitis hospitalisations for years 2017-2019 and socioeconomic deprivation as per the Index of Multiple Deprivation 2019 (see main paper).

The intervention was delivered mostly though established Children's Centre infrastructures, as well as across a range of family-convenient locations to ensure accessibility and maximum engagement. A significant portion of the intervention occurred at local Children's Centres, which provided a supportive infrastructure for parent-and-baby groups, health visitor clinics, and other activities. Individual sessions were conducted in participants' homes during post birth visits. Wider outreach efforts were carried out in key community hubs frequented by young families, including public libraries, nurseries, pharmacies, GP practices, schools, and local supermarkets.

When and how much:

The intervention combined recurrent sessions with regular attendees and one-off interactions tailored to families' needs:

Group Sessions: Parent Champions facilitated regular group sessions at Children's Centres, which were adapted to the needs of the community. These sessions often run weekly and provided ongoing education and peer support to families who attended consistently over several weeks or months.
One-Off Interventions: For some families, the intervention involved one-time interactions, such as during home visits, telephone calls, or larger community events. These sessions focused on immediate education, support, and signposting to resources.
Intensity and Duration: The intensity of engagement varied depending on the family's needs. Some families engaged with the intervention repeatedly over time through ongoing group or one-to-one sessions, while others received targeted, one-time support.

Tailoring:

The intervention was explicitly designed to be tailored at both the individual family level and the broader community level, ensuring it met the specific needs of local populations.

Tailoring at the Individual Family Level:
The support provided by Parent Champions was adapted based on the specific needs of each family. For example, families requiring additional support were referred to appropriate services such as poverty reduction initiatives or specialist organisations for housing, breastfeeding, or smoking cessation. Interactions were also adjusted to accommodate the family's preferences and circumstances, such as the mode of delivery (home visits, telephone calls, or group sessions).

Tailoring to Local Community Needs:
The intervention also adapted to the specific needs of the communities served, as identified by the Children's Centres and Parent Champions:
In communities with lower engagement in Children's Centres, more home visits and outreach sessions were conducted to reach families. Conversely, in communities with high engagement, the focus was on group sessions at the Centres.
In areas with high proportions of migrant families, Parent Champions supported English as a second language (ESL) classes and visited hotels housing asylum seekers to deliver targeted education and resources.

Tailoring of Training:
Training for Parent Champions was also adapted to meet the emerging needs they identified while working in their communities. Examples include: Immunisation education training to address vaccine hesitancy among families. Specific training on the newly launched RSV vaccine (September 2024 in the UK) to ensure Parent Champions were equipped to educate families about this intervention.

Modification:

The intervention underwent modifications during its implementation due to changes in funding and staffing:

Extension of the Intervention Duration:
The project was initially designed as a 6-month intervention funded by the NHSE VCSE fund. Due to its demonstrated success during this period, additional funding was secured to extend the intervention beyond its original timeframe.
Staffing Adjustments:
As the initial contracts were for only six months, four of the original nine Parent Champions chose not to extend their contracts, having already planned their commitments with the initial duration in mind. Three new Parent Champions were recruited shortly after the initial contracts ended to fill some of the vacancies. The newly recruited Parent Champions underwent the same training program as the original Parent Champions to ensure consistency in knowledge and skills across the team. For budgeting reasons, two Children's Centres involved in the initial phase of the intervention did not have their Parent Champion roles re-recruited.

These changes reflect both the challenges of sustaining pilot projects and the adaptability of the intervention team to continue delivering services.

How well (planned):

Delivery: The fidelity of the intervention's delivery was monitored using the Children's Centres' eStart system, which logged all interactions facilitated through Centre - coordinated sessions. This included both group sessions and individual home visits or consultations. Over the first two years of the intervention, Parent Champions recorded a total of 18,050 contacts using this system.

Reception: The reception of the intervention within the community was assessed through qualitative research conducted by Edge Hill University. This research included questionnaires and in-depth interviews with key stakeholders including the Parent Champions, Children's Centre leaders and parents who participated in the intervention. The intervention was received positively by stakeholders who "welcomed the opportunity to learn and improve the lives and health of their children" (1).
